# Quantifying Patient Portal Use: Systematic Review of Utilization Metrics

**DOI:** 10.2196/23493

**Published:** 2021-02-25

**Authors:** Lauren L Beal, Jacob M Kolman, Stephen L Jones, Aroub Khleif, Terri Menser

**Affiliations:** 1 Center for Outcomes Research, Houston Methodist Hospital Houston, TX United States; 2 University of Texas Health Science Center, McGovern Medical School Houston, TX United States; 3 Department of Surgery Weill Cornell Medical College New York, NY United States; 4 Ambulatory Clinical Systems, Information Technology Division, Houston Methodist Hospital Houston, TX United States

**Keywords:** patient portals, meaningful use, American Recovery and Reinvestment Act, Health Information Technology for Economic and Clinical Health Act, portal utilization, patient-generated health data, portal, systematic review

## Abstract

**Background:**

Use of patient portals has been associated with positive outcomes in patient engagement and satisfaction. Portal studies have also connected portal use, as well as the nature of users’ interactions with portals, and the contents of their generated data to meaningful cost and quality outcomes. Incentive programs in the United States have encouraged uptake of health information technology, including patient portals, by setting standards for meaningful use of such technology. However, despite widespread interest in patient portal use and adoption, studies on patient portals differ in actual metrics used to operationalize and track utilization, leading to unsystematic and incommensurable characterizations of use. No known review has systematically assessed the measurements used to investigate patient portal utilization.

**Objective:**

The objective of this study was to apply systematic review criteria to identify and compare methods for quantifying and reporting patient portal use.

**Methods:**

Original studies with quantifiable metrics of portal use published in English between 2014 and the search date of October 17, 2018, were obtained from PubMed using the Medical Subject Heading term “Patient Portals” and related keyword searches. The first search round included full text review of all results to confirm a priori data charting elements of interest and suggest additional categories inductively; this round was supplemented by the retrieval of works cited in systematic reviews (based on title screening of all citations). An additional search round included broader keywords identified during the full-text review of the first round. Second round results were screened at abstract level for inclusion and confirmed by at least two raters. Included studies were analyzed for metrics related to basic use/adoption, frequency of use, duration metrics, intensity of use, and stratification of users into “super user” or high utilizers. Additional categories related to provider (including care team/administrative) use of the portal were identified inductively. Additional analyses included metrics aligned with meaningful use stage 2 (MU-2) categories employed by the US Centers for Medicare and Medicaid Services and the association between the number of portal metrics examined and the number of citations and the journal impact factor.

**Results:**

Of 315 distinct search results, 87 met the inclusion criteria. Of the a priori metrics, plus provider use, most studies included either three (26 studies, 30%) or four (23 studies, 26%) metrics. Nine studies (10%) only reported the patient use/adoption metric and only one study (1%) reported all six metrics. Of the US-based studies (n=76), 18 (24%) were explicitly motivated by MU-2 compliance; 40 studies (53%) at least mentioned these incentives, but only 6 studies (8%) presented metrics from which compliance rates could be inferred. Finally, the number of metrics examined was not associated with either the number of citations or the publishing journal’s impact factor.

**Conclusions:**

Portal utilization measures in the research literature can fall below established standards for “meaningful” or they can substantively exceed those standards in the type and number of utilization properties measured. Understanding how patient portal use has been defined and operationalized may encourage more consistent, well-defined, and perhaps more meaningful standards for utilization, informing future portal development.

## Introduction

A patient portal is a secure online website, managed by a health care organization, that provides patients access to their personal health information [1-3]. Portals were developed to provide patients with a platform through which to claim ownership over their health care. For patients that adopt health care portals, usage of the portal has been shown to positively impact health outcomes [1]. Despite their introduction in the late 1990s to augment patient engagement [2], widespread adoption of patient portals was not seen until 2006 [2,4]. As of 2018, a reported 90% of health care organizations offer patients portal access, with the remaining 10% reporting plans to adopt this tool [5].

Numerous studies have investigated the relationship between patient portal utilization and health outcomes, specifically indicating a link between increased portal use and increased rates of patient engagement [6-9]. Notably, engaged individuals more actively participate in the management of their health care [10] and report enhanced patient satisfaction [11], a finding increasingly critical in patients with chronic diseases [12]. Patient portal utilization has been linked to “significant decreases in office visits…, changes in medication regimen, and better adherence to treatment” [13], along with improved chronic disease management and disease awareness [8,9]. Interestingly, even the content of patient messages was recently found to be associated with estimated readmission rates in patients with ischemic heart disease [14]. In these ways, patient portals have been cited as essential components of the solution to the cost and quality health care crisis in the United States [2].

A driving force behind the adoption and current progression of patient portals is the meaningful use (MU) criteria [13,15,16]. Introduced in 2009, the American Recovery and Reinvestment Act [2,16] included $30 billion [17] for the incentive program’s implementation to fund government reimbursements for patient-centered health care [13] with the goal of utilizing electronic exchange of health information to improve quality of care [2]. Specific program guidelines, including an emphasis on increasing patient-controlled data and financial incentives to interact with patients through a patient portal [1,18], resulted in increased portal utilization [1]. Interactive, MU-mandated features of patient portals currently include (1) a clinical summary following each patient visit, (2) support of secure messaging between the patient and health care provider, and (3) the functionality of viewing, downloading, and transferring patient data [2].

Coupled with advances in technology and continued movement toward focusing on patient-centered care, features beyond those described by MU criteria have been implemented, including online appointment scheduling and bill payments, and continue to shape portal evolution. Mirroring the benefits of this technology, numerical projections demonstrate that the rate at which patients wish to utilize patient portals far exceeds the rate at which this technology is provided to them by their health care providers [19], with an estimated 75% of individuals accessing their personal health records via patient portals by 2020 [19].

Despite widespread portal interest and adoption, as well as comprehensive reviews on patient engagement with portals [2], no review has systematically assessed measurements investigating patient portal utilization. Currently, measurement of patient portal use varies widely, with inconsistent conceptual definitions serving as a consistent limitation to robust analysis [20]. Understanding how patient portal use has been defined and operationalized, both previously and currently, will encourage consistent and well-defined utilization of patient portals. Further, standardization of patient portal measurements will provide a basis from which to systematically analyze how to continue developing patient portals best suited to consumer needs.

## Methods

### Study Eligibility Criteria

This systematic review includes original studies with quantifiable metrics of portal use, broadly construed. Subjective reporting on usability, design requirements, or other qualitative analyses were excluded as nontopical. Systematic reviews were also excluded, although their bibliographies were utilized for reference crawling. The criteria used to determine eligibility of studies employing self-reported use and prospective studies emerged inductively through interrater review and discussion (between TM, LLB, and JMK) based on preliminary results. Self-reporting measures were excluded unless they reported direct portal usage data that were quantifiable and similar to actual portal use tracking (eg, by frequency of logins, duration of sessions, number of functions used, etc). Prospective trial designs were omitted if they artificially influenced portal use but were included if the portal use metric could be reasonably abstracted from its experimental context (eg, as either a quantified outcome or an uncontrolled baseline measure).

Studies available in English, published between 2014 and the end search date of October 17, 2018, were eligible for inclusion. The year 2014 was selected due to the full rollout of Centers for Medicare & Medicaid Services (CMS) meaningful use (MU) stage 2 (MU-2) requirements and the emergence of “Patient Portals” as a Medical Subject Headings (MeSH) term (automatically including cognate terms “Patient Internet Portal,” “Patient Portal,” “Patient Web Portal,” and “Patient Web Portals,” and subsumed under “Health Records, Personal,” with previous indexing via the less focused term, “Electronic Health Records,” from 2010-2016). Although US MU-2 regulations were of particular interest, we did not exclude studies from other countries so that potentially informative use metrics employed outside the United States would also inform the results. Studies from outside the United States were not included in the analysis focused on MU.

### Identification and Selection of Studies

The search proceeded in two major rounds. In round 1, authors JMK and LLB identified studies in PubMed by applying the MeSH term “Patient Portals” with no other limiters. All round 1 results were initially reviewed in English full text if available rather than relying on title/abstract screening alone. Full texts were read to better orient the raters to the patient portal utilization literature, suggest secondary sources and search terms, confirm a priori data charting categories of interest (use/adoption, frequency, duration, intensity, and super user), and suggest additional charting elements inductively. Within the round 1 results, secondary searching was performed on all article bibliographies, and relevant titles were retrieved for review. Results prior to 2014 were excluded based on the emerging relevance and centrality of CMS MU.

After reviewing the literature (241 full-text articles from round 1, including 148 articles from PubMed and 93 articles from the bibliographic search), themes that emerged as commonly studies metrics became the basis of our coding for the complete two-part search process. These confirmed our a priori themes of interest and would serve as relevant limiters in the broader keyword-based second search to complement the more restrictive round 1 MeSH-based search. Round 2 searching (by TM) applied the following terms at title/abstract and keyword levels: “patient portal” AND (frequency OR use OR duration OR intensity). Duplicate results were removed by an automatic process using Stata matching on title, author, and year, followed by a manual check to remove additional duplicates that were missed (eg, formatting, punctuation, etc).

**Figure 1:**
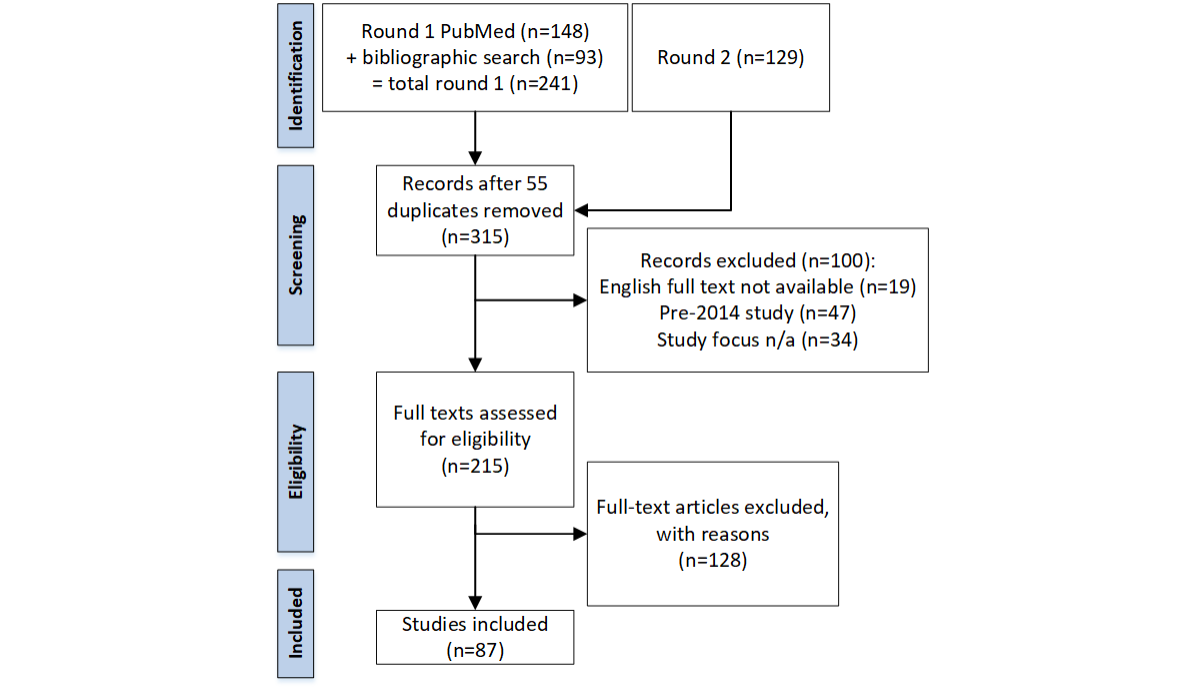
Study selection and inclusion process reported per guidelines of the Preferred Reporting Items for Systematic Reviews and Meta-Analyses (PRISMA) [21]. n/a: Not applicable.

The full results were screened at the abstract level to exclude non–English articles, those without full text, sources older than 2014, and articles lacking quantified portal metrics. Two raters (LLB and JMK) assessed inclusion at the abstract level, followed by full text review; any inclusion or data charting discrepancies were resolved in rater meetings (by LLB, JMK, and TM). Figure 1 summarizes the screening and inclusion process.

### Data Collection and Study Appraisal

For coding purposes, use/adoption, frequency, duration, intensity, and super user (or similar user stratification) were considered a priori themes from which to extract definitions; provider use emerged as a theme inductively. Super user, in this context, is synonymous with high utilizer and should not be confused with the information technology standard definition implying a user with elevated privileges. All metrics were coded as binary, indicating the presence of a measure for and/or definition of each respective metric. These data were coded and recorded in a spreadsheet containing the article citation information and columns for themes of interest for both portal use metric definitions and MU criteria. Extractors’ working definitions of metric types are summarized in Table 1.

**Table 1:** Study inclusion metric definitions.

Metric	Definition used for data charting
Provider use	Portal use by providers, care teams, or other staff. This use could be in terms of adoption, frequency, intensity, duration, or super user, per below; patient utilization grouped by provider practice/specialty also implies provider/practice adoption.
Patient use/adoption	Patient use/adoption, including proxies, parents, or surrogates acting on behalf of patients. Total number/percentage of registrations, logins, or other basic access information falls under this category.
Frequency	Metrics that involve count variables per time unit or within a study window (eg, patient logins per week, message rates per month, functions used during an inpatient window).
Duration	Duration as a continuous time variable (eg, an average patient login session time of “n” minutes, months/years during which a user account remained active, time from visit to first login); “length” variables for message threads (two vs three messages per thread) were also interpreted as a type of duration, although only in one instance.
Intensity	Counts that include a qualitative “depth” component beyond clicks or page accesses, including counts of messages with clinical relevance, message threads leading to clinical resolution, and use of functions entailing substantive input/data entry, such as diaries or care preference plans.
Super user	Users stratified in a way that distinguishes a high-utilization or high-activity group (eg, in terms of greater intensity, a categorically higher frequency, consistent duration of use, etc).

For included articles from the United States, a set of CMS MU themes emerged as relevant and were added upon agreement by all raters. An “MU motive” coding tag indicated either explicit desire for MU compliance in the article or at least mention of MU (eg, in the introduction or discussion). “MU consistent” tags rated apparent compatibility between the metric used and basic MU-2 requirements (regardless of motive), and included (1) “access”—coded if the percentage of patients accessing the portal information could be derived; (2) “send”—coded if patient-initiated messaging was tracked; (3) “view-download-transmit” (VDT)—coded if the percentage of patients who could VDT (specifically within the 4-day window following an office visit or 36 hours after discharge from hospital) could be determined from the metrics reported; and (4) MU-2—coded as shorthand when all three conditions, which together entail the full MU-2 compliance requirements, were met.

Finally, in lieu of assessing the methodological quality of these wide-ranging patient portal studies, we assessed quality based on two criteria: the quality of the journals in which articles were published based on their 2019 impact factor (except in one instance where the 2018 impact factor was used), and the citation count of each article. For the latter, we extracted the total number of citations (as of September 22, 2020) to calculate the mean number of citations per year that each article received based on the time period that elapsed since online publication date. Regression analyses were conducted to determine if the number of use metrics was predictive of the mean number of citations per year or the impact factor of the journal in which the article was published.

## Results

A total of 315 search results remained after the removal of duplicates. All 315 articles were examined for defined patient portal metrics, with records excluded (n=100) for the following reasons: lack of the full-text English-language article or a suitably detailed abstract (non–English-language [n=18] or no text available [n=1]), study publication date prior to 2014 (n=47), and/or nonapplicable study focus (n=34). The remaining 215 studies were analyzed; of these, 128 were excluded, leaving 87 studies for inclusion in the analysis (see Figure 1). Notably, the abstracts (or translations thereof) of 18 non–English-language exclusions also met other exclusion criteria (eg, qualitative only/no metrics defined, portal development or usability studies, or literature reviews of unrelated portal topics).

Patient use was the most commonly studied patient portal metric, analyzed in 90% (78/87) of studies. Super user designations were only found in 24% (21/87) of studies, making this the least commonly studied metric. Table 2 identifies the frequency with which each metric was included in each study, with totals for each metric [6-10,18,22-102]. There were 32 different combinations of study metrics, identified in Table 3, with the two most common metric combinations being patient use/adoption, frequency, and intensity (n=9) and patient use/adoption alone (n=9). The majority of studies (53/87, 61%) analyzed three or fewer metrics, with 3.11 as the average number of metrics reported. The definitions of these 271 metrics are summarized by study in Multimedia Appendix 1.

**Table 2:** Frequencies of metric inclusion in analyzed studies (N=87).

	Metric^a^					
Study	Provider use	Patient use/adoption	Frequency	Duration	Intensity	Super user
Ackerman et al, 2017 [60]	✓^b^	✓	✓	✓	✓	
Ahmedani et al, 2016 [92]		✓	✓			
Aljabri et al, 2018 [69]		✓			✓	
Alpert et al, 2017 [98]	✓		✓	✓	✓	✓
Arcury et al, 2017 [47]		✓	✓		✓	
Bajracharya et al, 2016 [89]		✓	✓		✓	
Baldwin et al, 2017 [83]		✓				
Bell et al, 2018 [50]		✓	✓		✓	
Boogerd et al, 2017 [31]	✓	✓^c^	✓			✓
Bose-Brill et al, 2018 [67]	✓		✓		✓	✓
Bower et al, 2017 [93]		✓				
Chung et al, 2017 [70]	✓	✓^c^	✓		✓	✓
Crotty et al, 2014 [58]	✓	✓	✓		✓	
Crotty et al, 2015 [100]	✓		✓	✓	✓	
Dalal et al, 2016 [101]	✓	✓^c^	✓		✓	
Davis et al, 2015 [96]		✓			✓	
Devkota et al, 2016 [51]		✓			✓	
Dexter et al, 2016 [77]		✓				
Emani et al, 2016 [30]		✓	✓	✓	✓	
Fiks et al, 2015 [68]			✓			✓
Fiks et al, 2016 [53]		✓			✓	
Forster et al, 2015 [22]		✓	✓	✓	✓	
Garrido et al, 2014 [39]	✓	✓	✓		✓	✓
Garrido et al, 2015 [23]		✓				
Gheorghiu and Hagen, 2017 [24]		✓	✓			
Gordon and Hornbrook, 2016 [62]		✓			✓	
Graetz et al, 2016 [86]		✓	✓			
Griffin et al, 2016 [18]		✓	✓		✓	✓
Groen et al, 2017 [49]		✓	✓	✓		
Haun et al, 2014 [73]	✓	✓			✓	
Henry et al, 2016 [46]		✓				
Jhamb et al, 2015 [44]		✓	✓		✓	
Jones et al, 2015 [71]		✓	✓	✓	✓	✓
Kamo et al, 2017 [56]		✓	✓	✓	✓	
Kelly et al, 2017 [72]		✓	✓		✓	✓
King et al, 2017 [34]	✓	✓	✓	✓	✓	
Kipping et al, 2016 [25]		✓			✓	
Krasowski et al, 2017 [36]	✓	✓		✓		
Krist et al, 2014 [61]		✓	✓	✓		
Laccetti et al, 2016 [41]	✓	✓	✓		✓	✓
Lau et al, 2014 [29]		✓				
Lyles et al, 2016 [9]		✓^c^	✓		✓	✓
Mafi et al, 2016 [45]		✓	✓	✓		
Manard et al, 2016 [35]		✓			✓	
Masterman et al, 2017 [99]	✓				✓	
Masterson Creber et al, 2016 [7]			✓	✓	✓	
Mickles and Mielenz, 2014 [78]	✓	✓	✓	✓	✓	
Miles et al, 2016 [82]		✓^c^				
Mook et al, 2018 [74]		✓				
Neuner et al, 2015 [42]	✓	✓	✓	✓	✓	✓
North et al, 2014 [102]		✓^c^	✓	✓		✓
Oest et al, 2018 [80]		✓			✓	
Payne et al, 2016 [84]	✓	✓				
Pearl, 2014 [38]	✓	✓		✓	✓	
Pecina et al, 2017 [54]	✓	✓^c^	✓			
Peremislov, 2017 [91]	✓	✓^c^		✓	✓	
Perzynski et al, 2017 [63]		✓	✓		✓	
Petullo et al, 2016 [75]		✓	✓		✓	
Phelps et al, 2014 [52]		✓	✓	✓		✓
Pillemer et al, 2016 [37]	✓	✓			✓	
Price-Haywood and Luo, 2017 [6]		✓				
Quinn et al, 2018 [97]		✓^c^	✓		✓	
Redelmeier and Kraus, 2018 [26]		✓	✓		✓	
Reed et al, 2015 [57]	✓	✓	✓			
Reicher and Reicher, 2016 [64]		✓		✓	✓	
Riippa et al, 2014 [27]		✓	✓		✓	✓
Robinson et al, 2017 [81]		✓	✓		✓	✓
Ronda et al, 2014 [48]		✓	✓			
Runaas et al, 2017 [28]	✓		✓	✓		
Sarkar et al, 2014 [8]		✓^c^			✓	✓
Shaw et al, 2017 [65]	✓	✓		✓	✓	
Shenson et al, 2016 [76]	✓	✓	✓			
Shimada et al, 2016 [95]		✓^c^	✓	✓	✓	
Smith et al, 2015 [85]	✓	✓	✓		✓	
Sorondo et al, 2017 [43]		✓	✓		✓	
Steitz et al, 2017 [87]		✓	✓	✓	✓	
Thompson et al, 2016 [32]	✓	✓			✓	
Toscos et al, 2016 [40]			✓		✓	✓
Tulu et al, 2016 [10]	✓	✓			✓	
Vydra et al, 2015 [90]	✓	✓	✓	✓	✓	
Wallace et al, 2016 [88]		✓	✓		✓	✓
Weisner et al, 2016 [66]			✓		✓	
Williamson et al, 2017 [55]		✓	✓	✓	✓	
Wolcott et al, 2017 [59]	✓	✓^c^	✓		✓	✓
Wolff et al, 2016 [94]		✓			✓	
Woods et al, 2017 [33]		✓	✓			✓
Zhong et al, 2018 [79]		✓		✓		
Total	30 (34%)	78 (90%)	56 (64%)	27 (31%)	59 (68%)	21 (24%)

^a^See Multimedia Appendix 1 for full definition of each metric from each article.

^b^Indicates presence of the metric.

^c^Special contexts shaped the form of the metric in ways atypical of direct use analysis (eg, due to experimental controls).

MU serves as a driving criterion for patient portal adoption and utilization, reflected by the 87% (66/76) of US publications that included MU criteria, irrespective of an explicit motive, and 24% (18/76) of US studies explicitly implicating MU criteria as a driving force behind their publication. However, 33% (29/87) of the total manuscripts did not include any MU motive, whether because of foreign publication (n=11) or, among the 76 US sources, lack of statement of MU motivation (n=17) or a statement that the data and analysis did not fit the MU criteria because the study conduct predated its release (n=1). These studies indicate that investigation of metrics surrounding portal utilization extends beyond MU motives and metrics. Lau and colleagues [29] mentioned that while exact login time stamps were noted to be available, frequency, duration, and intensity metrics went unanalyzed in favor of a simplified metric. Similarly, the study conducted by Emani et al [30] mentioned MU stage 1 requirements, yet researchers purposefully extended the CMS 3-day window to 5 days for patients to access their postvisit summary. Further, Boogerd and colleagues [31] analyzed portal implementation through portal inaccessibility; this was achieved through measuring login difficulties and downtime while stratifying implementation efficacy through self-reported parenting stress, an efficacy measure not widely seen in the published literature. Mirroring Boogerd, Thompson et al [32] included information regarding the number of patient and proxy portal password changes, and Woods et al [33] reported on the number of unsuccessful or incomplete logins per study subject. Thus, several studies examined meaningful metrics of portal use and usability exceeding, or at least not anticipated by, MU-2 requirements. These metrics reflect portal utilization beyond the criteria of MU.

The patient use/adoption metric was the most frequently studied of the analyzed variables, included as a study metric in 90% (78/87) of studies (Table 2). Comparatively, provider use was analyzed in only 34% (30/87) of studies and rarely studied without simultaneously investigating patient use/adoption (5/87, 6%). Teasing apart patient use from provider use is an important distinction; however, some studies combine these distinct data points together and analyze the summed use [34]. Mirroring this, other studies group together patients not registered for the portal with registered patients that haven’t messaged, highlighting the variability in reported metrics [35]. Similarly to the definitions of other analyzed metrics, provider use definitions revealed variability: while Krasowski et al [36] and Pillemer et al [37] tabulated provider use through “manual release of test results ahead of automatic release,” others calculated this metric through provider response to patient messages [38,39]. The combined analysis of patient and provider use continues in more recent literature. Margolius et al [103] found that having a wealthier or larger patient population and working more days per week resulted in primary care physicians receiving more messages from patients, which the authors stratified into message types. Provider use has been shown to lead to patient use [20], but while patients can be led toward engagement with a system required by their physicians, they can also be led away from a system not utilized by their providers [104] (eg, if patients message their providers but don’t receive a response). As mentioned previously, patient portal utilization has been employed as a proxy for patient engagement, with increased portal usage associated with better patient outcomes [105].

Notably, of the investigated metric groupings seen in Table 3, 59 studies (68%) included intensity as an analyzed metric, signaling the perceived importance of the depth at which patients were engaging with the portal. The definitions utilized by these studies varied: Emani and colleagues [30] distinguished between portal sessions and portal message use; Toscos et al [40] included intensity as the proportion of users engaging the daily health diary function; and Laccetti et al [41] investigated “staff MyChart actions performed per patient-initiated message.”

**Table 3:** Number of metrics and metric combinations analyzed in 87 studies.

Number of metrics analyzed	Studies analyzing stated metrics, n (%)	Metric combinations
1	9 (10)	Patient use/adoption (n=9)
2	18 (21)	Patient use/adoption + intensity (n=9) Patient use/adoption + frequency (n=4) Patient use/adoption + duration (n=1) Provider use + patient use/adoption (n=1) Provider use + intensity (n=1) Frequency + intensity (n=1) Frequently + super user (n=1)
3	26 (30)	Patient use/adoption + frequency + intensity (n=9) Patient use/adoption + frequency + duration (n=3) Patient use/adoption + provider use + intensity (n=4) Patient use/adoption + provider use + frequency (n=3) Frequency + intensity + super user (n=1) Provider use + frequency + duration (n=1) Frequency + duration + intensity (n=1) Patient use/adoption + intensity + super user (n=1) Provider use + patient use/adoption + duration (n=1) Patient use/adoption + duration + intensity (n=1) Patient use/adoption + frequency + super user (n=1)
4	23 (26)	Patient use/adoption + frequency + intensity + super user (n=6) Patient use/adoption + frequency + duration + intensity (n=6) Provider use + patient use/adoption + duration + intensity (n=3) Provider use + patient use/adoption + frequency + intensity (n=3) Patient use/adoption + frequency + duration + super user (n=2) Provider use + frequency + intensity + super user (n=1) Patient use/adoption + provider use + frequency + super user (n=1) Provider use + frequency + duration + intensity (n=1)
5	10 (11)	Provider use + patient use/adoption + frequency + intensity + super user (n=4) Provider use + patient use/adoption + frequency + duration + intensity (n=4) Patient use/adoption + frequency + duration + intensity + super user (n=1) Provider use + frequency + duration + intensity + super user (n=1)
6	1 (1)	Provider use + patient use/adoption + frequency + duration + intensity + super user (n=1)

**Figure 2:**
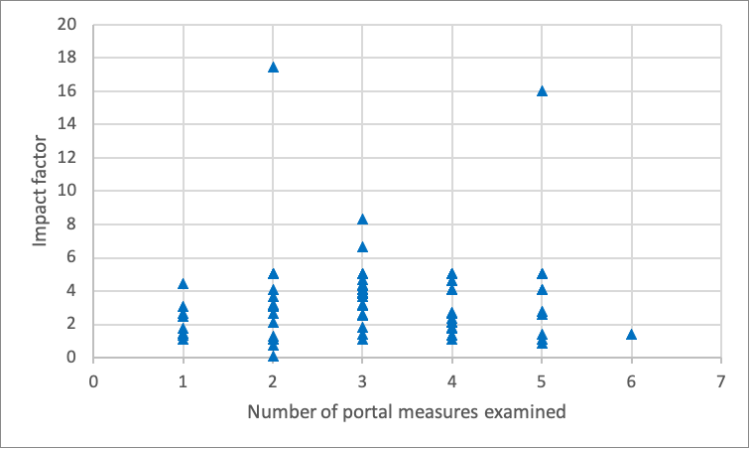
Relationship between the number of portal measures examined in an article and the impact factor of the publishing journal.

**Figure 3:**
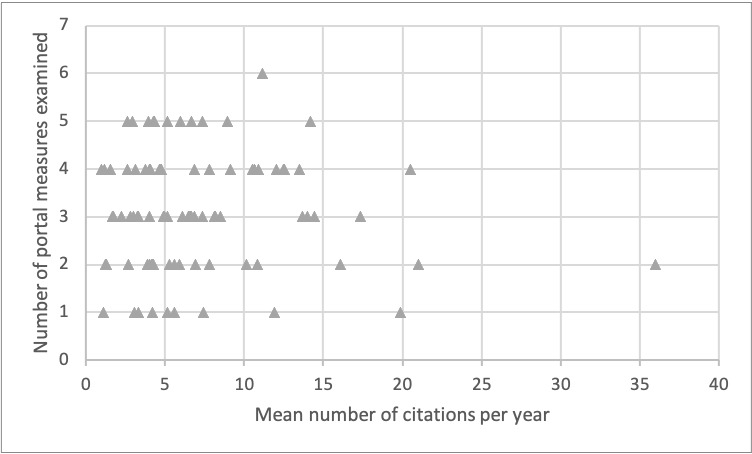
Relationship between the number of portal measures examined in an article and the mean number of citations per year (via Google Scholar).

Neither of the quality variables (ie, journal impact factor and citation count) were shown to be statistically significantly associated with the number of patient portal metrics described in Table 3. The relationship between the number of patient portal metrics examined and the journal impact factor and the mean number of citations per year are visually depicted in Figures 2 and 3, respectively.

Table 4 depicts studies that were consistent with some metric of MU criteria. Articles not published in the United States were excluded from MU analysis due to variability in portal guidelines by country. Combining the three MU metrics (ie, access, send, and VDT), 10 studies (13%) out of the 76 studies conducted in the United States did not meet at least one of the MU metrics, meaning that 66 studies (87%) did analyze MU criteria in some capacity, irrespective of an explicit motive. However, only 18 (24%) of the 76 US studies explicitly implicated MU criteria as a driving force behind their study.

Further data analysis revealed that a larger percentage of manuscripts investigated three (30%) or four (26%) metrics rather than two metrics (21%), nodding to the perceived complexity of the relationship between variables influencing portal utilization. Only one study, by Neuner and colleagues [42], investigated all six variables included in MU guidelines, focusing on investigating enrollment and use based on MU guidelines, as well as satisfaction, highlighting the lack of exhaustive analysis of all available metrics. While “use” in this manuscript was defined as patients accessing their patient portal, use in other studies has been defined as number of enrollees [32], access plus protocol-specific assessments and secure messaging [43], percentage with at least one login [44], and number of patients who viewed physician notes within 30 days of their visit [45]. While Henry and colleagues [46] defined use as registration versus not, Arcury et al [47] and Graetz et al [86] analyzed use as a patient-reported binary metric, highlighting the variability in this metric’s composition. The many studies stratifying use based on at least one login could be capturing the login required to create the account and not portal utilization as proxy for health care engagement [6,29,48-50]. Some of these studies created specific classifications for users, including “nonusers,” “readers,” and “readers and writers” [51], potentially to mitigate their definition of use. As an example of the complexity of patient portal data, in an effort to ensure accuracy of the study population, Phelps et al [52] stratified users by the absence of any portal login in the past 6 months, despite at least two lab uploads, to ensure the population studied was alive and had reason to access the portal. Others classified use by the completion of at least one survey during the study period [53], the total number of patients on the mobile app [38], the number of patients initiating the online refill function [8], or contact with the care messenger via the portal [54], highlighting the variability in the definition of this fundamental metric.

**Table 4:** Meaningful use (MU) definitions in US studies (n=76).

Measure	Definition	Studies with requisite measure, n (%)^a^	Study citations
Explicit MU motive	Authors state outright that the motivation behind the study stemmed from MU criteria	18 (24)	[7,18,30,32,37,42,45,55-65]
Mention of MU	Some mention of MU was made in the introduction or discussion	40 (53)	[6,8-10,33,36,38-40,43,44,47,50,53,54,66-90]
Access	Patient login into the portal	45 (59)	[6,9,18,30,32,33,36-38,40,42-45,50,51,53-56,58,60-66,69,71,72,80-82,85,87-96]
Send	Sending a secure message to the health care provider	45 (59)	[8-10,18,30,32,35,39,40,42-44,50,51,54-60,62,63,65-67,70-73,75-78,85-88,91,94-99]
View-download-transmit (VDT)	Ability to view, download, and transmit health information within 4 days of an office visit (providers) or 36 hours of discharge (hospital)	9 (12)	[36,42,56,60,61,65,72,81,96]
MU-2	Met requirements for access, send, and VDT	6 (8)	[42,56,60,65,72,96]

^a^Percentages exceed 100% total because studies could meet more than one criterion, and MU-2 represents studies that met all three conditions (access, send, and VDT) that together entail the full requirements of Centers for Medicare & Medicaid Services MU stage 2.

Lacking standardized definitions, variable consistency in the application of MU terminology appears throughout the published literature [2,32,43,106]. At the most fundamental level, the lack of distinction between the patient health record (PHR), whose ownership and management lies with the patient, and the patient portal, whose ownership and management lies with the health care organization, was evident in publications that investigated patient portals but included information on PHRs in the statistical analysis [13,104,107]. Further, Devkota and colleagues [51] highlighted that mixed outcomes regarding the relationship between frequency of portal utilization and health outcomes are rooted in how studies analyze portal interaction; while some studies focused on message counts [31,41,70], others focused on interaction intensity with providers through portal messaging, stratifying by no use, read-only, and read-and-write [18,43]. Further, Jones et al [71] included consistency as part of their frequency measurement, an inclusion not found in other manuscripts. Baldwin and colleagues [83] explicitly stated in their manuscript that “registration rates and ID verifications do not account for the people who register but do not actively use the portal,” citing difficulties in their “use” analysis from patients who “report login issues and difficulty navigating portals.”

## Discussion

### Principal Findings

Portal analyses have extended beyond MU-2 criteria in an effort to best meet provider and patient needs. Fast Pass—an “automated rescheduling system” requiring opt-in through a patient portal—not only indirectly measured patient use/adoption through logins to enter the program but showed that automated rescheduling prompts reduced no-show appointments by 38% [108]. Patient portal utilization extending beyond MU criteria has been critical during the COVID-19 pandemic, with Patel et al [109] describing how pediatric patient portals pivoted from traditional, in-person enrollment methods and Judson et al [110] detailing the creation of a COVID-19 self-triage and assessment tool for primary care patients. The widespread patient portal adoption in the United States provides the necessary foundation for patients to access telemedicine visits while simultaneously creating a digital divide—a topic vastly cited since the emergence of patient portals [111-113]. Graetz et al [86] asserted that the digital divide particularly impacts disadvantaged groups; they observed that the use of a personal computer and internet access “explained 52% of the association between race and secure message use and 60% of the association between income and use,” and suggested that providing portal access across multiple platforms, including telephones, could reduce message use disparities. Further, initiation of portal use has been found to be “lower for racial and ethnic minorities, persons of lower socioeconomic status, and those without neighborhood broadband internet access,” leading to a digital divide in portal utilization [63]. A mismatch between “MU-based metrics of patient engagement and the priorities and needs of safety net populations” has also been cited [60], mirroring the recent combination of patient and provider use by Margolius et al [103] that found an increased quantity of patient-driven messages received by clinicians with a more robust or wealthier patient base. We recognize that portal utilization varies widely across institutions, with some institutions using patient portals for appointment scheduling, uploading demographic data and completing assessments prior to clinical visits, and even downloading parking passes for on-site visits, while other institutions emphasize portal use more heavily for message utilization and/or lab results. While our investigation was focused primarily on definitional differences in use across institutions, future investigations should explore portal-specific patient education and training, along with differences in portal functionality.

Recognizing that the intended target/purpose of portal interventions varies widely, it follows that the patient portal metrics utilized will be based on the functionalities being tested in each intervention. This fact results in the inability to generate specific overarching recommendations regarding portal analysis; however, systematic analysis of portal functions using clear definitions provides a foundation from which future studies can more readily compare portal use. Further, defining “use” more substantively by removing the baseline of single login—which could be the login used to create the portal itself without meaningful interaction with portal functions—could further facilitate the generation of meaningful utilization data. Therefore, we recommend that future studies clearly and specifically define the portal metrics utilized to allow for comparisons across studies and avoid using a binary measure of patient use/adoption that includes just one login, as the creation of a portal account often requires an initial login that does not necessarily equate to any MU. Relatedly, Gheorghiu and Hagens [106] criticized analyzing only the aggregate number of portal accesses because this cannot distinguish between a large yet infrequent number of users from a few frequent users. Further, we recommend that all future patient portal studies include the following population characteristics: the total organization population that could have access to the portal; the number of patients (and percentage of total) that currently have a patient portal account (regardless of use); and the number of patients that have used their account within the past year, with use being defined as two logins. This information will allow for meaningful comparisons across studies without being overly cumbersome to attain.

The diversity of metrics found in this review may also inform patient portal operations and dashboards of what may be worth tracking for research purposes. Additionally, the categories that emerged during this review could be used going forward to classify the variables of interest in future patient portal studies (ie, patient and/or provider use/adoption, duration, frequency, intensity, and super user). A few articles noted in the study limitations that a metric the authors deemed valuable to report could not be tracked for lack of available data. These included data on frequency for early adopters [84], intensity in the form of portal components accessed [35], and patient access to radiology images—the importance of which the authors noted was independent of MU-2 compliance, but, being unrelated to compliance, was not available [64]. One cannot study—or improve—what one does not track or offer to patients.

Further, the stratification of patient use/adoption provides an important area for future analysis. For instance, Zhong et al [79] cited the lack of quantification of active use (eg, in terms of per-user frequency or by-function intensity) as a study limitation. Some studies analyzed portal utilization through more “active” measures using a variety of multicomponent or stratified criteria. For instance, Devkota et al [51] grouped patients into “nonusers” who either did not activate their account or activated the account but did not write a message, “readers” who accessed but did not reply to emails, and “readers and writers” who read and subsequently wrote emails [51]. Oest et al [80] delineated commonly accessed portal features, including access to outpatient laboratory and radiology results. Miles et al [82] stratified use by types of available reports accessed—that is, the percentage of patients who viewed their radiology results were compared with views for other reports among all portal-registered patients. Manard et al [35] delineated active versus no active use by patients who wrote messages versus those who did not register or registered but only read messages.

Examples of methods used to stratify patient use/adoption in more “passive” terms include grouping patients by login versus no login (eg, Ronda et al [48] and Price-Haywood and Luo [6]) and defining use as patients registered for notifications versus those not registered at all (Henry et al [46]). Jones et al [71] defined an “active user” as one who had at least two portal sessions over the study period, with session defined as login-to-logout or until the 20-minute timeout occurred. Mirroring this, Petullo et al [75] distinguished account activation, defined as “active,” from patients sending messages, defined as “users.” Both Jones et al [71] and Petullo et al [75] created an interesting dynamic in which the publication employed the terminology of “active” without necessitating further portal engagement beyond login. Further, Masterson Creber et al [7] defined their own “Patient Activation Measure” to gauge patient engagement with portal functions, highlighting the need to more concretely define the parameters surrounding “active” versus “passive” portal use (and to disambiguate “active” as in user activity from merely “active” as in activated/registered accounts of otherwise passive users). Future measures should attempt to create a distinction between active and passive use.

Provider portal utilization drives patient utilization, with provider messaging levels and types predicting subsequent patient communication behavior [56] and provider responses to other patients’ messages driving a statistically significant increase in messages initiated by their patients [59]. A total of 30 studies in our systematic review specifically analyzed provider use, with nearly half of those studies analyzing provider-initiated messages. This fact further highlights the notion of physician use predicting patient use of portal functions and the intersection of physician portal use with institutional support of portal utilization. Recognizing that organizational policies mandate physician portal use, and nonuse, future studies should examine metrics for provider use more distinctly from patient use. As mentioned previously, provider use was only analyzed separately from patient use in 6% of studies, generating an untapped lens through which to investigate the driving forces of patient portal utilization. Mafi et al [45] found that individualized physician reminders to patients alerting them of completed visit notes drove patient portal utilization and engagement. However, provider and patient utilization are also intrinsically linked: Laccetti et al’s [41] analysis on portal use by clinical staff hinged on the clinical actions performed based on received patient messages, and Crotty et al’s [100] analysis of characteristics of unread messages necessitated message sending by the provider and lack of message reading by the patient. A recent study by Huerta et al [105] provided guidance on patient portal log file analysis and developed a taxonomy of computed analytic metrics. Patients who utilize their portal for longer periods are more likely to prefer communication through said portal, highlighting the importance of analyzing both patient and provider utilization [114].

### Conclusion

Our investigation supports the claim that not all health care systems study patient portal utilization systematically; thus, health care system support of different communication modalities is essential. Currently, the published literature is limited to analysis that is mostly based on patient portal utilization, as defined by MU criteria. More in-depth studies, mirroring the log file analysis conducted by Huerta et al [105] and, more recently, Di Tosto et al [115] that included a blueprint of individual patients’ portal actions, would fulfill our endeavor to utilize patient portal data more completely than the literature currently reports routinely. A systematic approach to measurement of portal usage is necessary to more readily draw comparisons across existing and future studies. Investigation of both provider and patient use/adoption will provide insight to generate a platform that is most beneficial for all users. One important limitation to note is that our review was limited to one database, but the main outlets for patient portal studies were included. Further, this is the largest known review examining patient portal research and the only review focusing on associated MU compliance assessment. Future investigation should more holistically analyze patient portal components in combination with the utilization of health services to elicit potential relationships currently unseen between portal use and patient health outcomes and to explore use that is, in the given context, truly meaningful.

## Appendix

Multimedia Appendix 1 Detailed summary of included studies.

## Abbreviations

CMS: Centers for Medicare & Medicaid Services
MeSH: Medical Subject Heading
MU: meaningful use
MU-2: Centers for Medicare & Medicaid Services meaningful use stage 2
PHR: patient health record
VDT: view-download-transmit
